# Is cross-sectional imaging necessary for fractures of the distal lower leg in children and adolescents: results of a nationwide survey

**DOI:** 10.1007/s00068-023-02379-6

**Published:** 2023-10-23

**Authors:** Jonas Alexander Strohm, Ilona Schubert, Dorien Schneidmüller, Peter Christian Strohm

**Affiliations:** 1 Department of Orthopedic and Trauma Surgery, University of Freiburg Medical Center, Hugstetter Str. 55, 79106 Freiburg, Germany; 2 Clinic for Orthopedics and Trauma Surgery, Klinikum Bamberg, Buger Straße 80, 96049 Bamberg, Germany; 3 Department of Trauma Surgery, BG Unfallklinik Murnau, Prof.-Küntscher-Str. 8, 82418 Murnau Am Staffelsee, Germany

**Keywords:** Children, Adolescent, Ancle fracture, Distal lower leg, Cross-sectional imaging

## Abstract

**Purpose:**

In childhood and adolescence, cross-sectional imaging, most commonly computed tomography (CT), is often performed for advanced diagnosis of joint injuries of the distal lower leg and upper ankle. Due to radiation exposure, the need for CT remains controversial, as these injuries follow stereotypies and usually have a similar course. Alternatively, the performance of magnetic resonance imaging (MRI) is also discussed. Since radiation sensitivity at this young age is much higher than in adults, an effort must be to minimize radiation exposure according to as low as reasonably achievable (ALARA) principles. The aim of this survey is to evaluate the current procedure in Germany in the diagnosis of pediatric injuries of the distal lower leg and upper ankle.

**Methods:**

For data collection, a survey entitled “CT in fractures of the ankle joint in childhood and adolescence: subject of the survey are injuries between 8 and 15 years of age” of the Section of Pediatric Traumatology in the German Association of Trauma Surgery was sent to all members via the distribution list of the German Society of Orthopedics and Traumatology and the distribution list of the German Society of Pediatric Surgery in a period from September 20, 2022–December 21, 2022. The survey included a total of 21 questions. Target groups were trauma and pediatric surgeons and orthopedic surgeons working in the hospital and in practice.

**Results:**

A total of 525 participants took part in the survey: ultrasound diagnostics are used by almost 25% and the Ottawa Ankle Rules by over 50% always or in most cases. A conventional x-ray is always or most often used by over 90%. CT imaging is rarely used by 88.57%, mainly for surgical planning or analysis of fracture progression. 69.9% report that their radiology department uses a pediatric protocol for CT exams; 25.71% do not know if this is the case. MRI imaging is also used infrequently by 89.33%, mostly to identify associated injuries. Overall, CT imaging is chosen by 55.62% and MRI imaging by 35.24% as the sectional imaging modality for suspected fractures; 95.05% consider sectional imaging useful for a triplane fracture, 59.24% for a two-plane fracture, 41.71% for a Salter-Harris type III/IV injury, and 8% for a Salter-Harris type I/II injury.

**Conclusion:**

The survey showed that the conventional X-ray is still the gold standard. Interestingly, more than half of the respondents regularly use the Ottawa Ankle Rules, and diagnostics using ultrasound are also used by almost a quarter. Awareness of radiation protection in children exists, although a quarter of all participants do not know the extent to which their radiology department has a specific pediatric protocol for CT imaging. Cross-sectional imaging is performed on a regular basis. Regarding the actual extent of imaging, there is a clear divergence between theory and practice.

## Purpose

Pediatric ankle injuries are among the more common injuries in childhood and adolescence, accounting for approximately 5% of all pediatric fractures. They can involve both the meta- and epiphyses of the distal tibia and fibula and account for the largest proportion of all lower extremity epiphyseal injuries, nearly 15–20% [1, 2]. Fracture patterns differ primarily because of the maturity of the growth plate [3]. This also causes a double incidence in boys compared to girls, mainly due to the later closure of the growth plates [1]. In contrast to adults, infantile ankle injuries more frequently show bony avulsions or fractures in the area of the growth plates due to the more stable ligamentous structures [4].

While conventional radiography remains the standard of care in imaging pediatric fractures, sonography has also become increasingly important in pediatric fracture diagnosis in recent years. Advantages of this imaging, in addition to the lack of radiation exposure and an unrestricted and rapid availability (both in the practice and in the hospital), are the fact that the injured children can remain on the arm or lap of the parents and the injured limb can be examined in a more comfortable gentle position for them [5]. However, sonography has not yet established itself as a standard diagnostic method, and the study situation is not yet as good for the lower leg as it is, for example, for the distal forearm [6].

Especially in pediatric joint injuries of the distal lower leg or upper ankle, respectively, cross-sectional imaging is often used for advanced diagnosis and planning of surgical care, most commonly computed tomography (CT). Despite special radiological protocols for children, this is associated with high radiation exposure, which must be avoided as much as possible, especially in children, due to their increased sensitivity to radiation. Here, the as low as reasonably achievable (ALARA) principles should always be followed [7]. Therefore, the use of MRI is also discussed as an alternative [8, 9].

The aim of this survey is to assess the current practice in Germany in the diagnosis of pediatric injuries of the distal lower leg and the upper ankle joint.

## Methods

For data collection, an online survey entitled “CT in fractures of the ankle joint in childhood and adolescence: subject of the survey are injuries between the ages of 8 and 15 years” of the Section of Pediatric Traumatology (SKT) in the German Association of Trauma Surgery (DGU) was sent to all members via the distribution list of the German Society of Orthopedics and Traumatology (DGOU) and the distribution list of the German Society for Pediatric Surgery (DGKCH) during a period from September 20, 2022–December 21, 2022. The survey included a total of 21 questions, 16 of which allowed only a single answer, 5 allowed multiple answers. The target group was trauma and pediatric surgeons and orthopedic surgeons working in hospitals and practices. The survey was conducted using the SurveyMonkey program (Momentive Inc., San Mateo, California, USA, www.momentive.ai) via the DGOU portal. In addition to questions about epidemiological data such as training level and field of practice, the survey asked about standards and possibilities in the respective clinics/practices as well as about personal diagnostic procedures and assessments. Finally, the further diagnostic procedure for different infantile ankle fractures was inquired on the basis of concrete X-ray images.

The results of the survey were displayed as anonymized data in Excel format. Further processing and analysis of the data was performed using the programs Filemaker and JASP. Here, in addition to absolute data, the percentage distributions were also determined.

## Results

A total of 525 participants took part in the survey over a period of 3 months. Of these, 15.05% reported working in a medical practice and 84.95% work in a hospital. 4% of the respondents are working as a resident, 16.95% as a specialist, 46.29% as a senior physician, and 32.76% as a chief physician. When asked about their (desired) specialty qualification, 88.43% indicated orthopedic and trauma surgery, 9.22% pediatric surgery, and 2.35% other specialty. Further, respondents work in nearly equal proportions in a local (21.9%), regional (25.33%), and national trauma center (26.67%). 15.05% work in private practice, and pediatric surgery and (pediatric) orthopedics are represented by 6.86% and 4.19%, respectively.

The question about the available imaging facilities in the practice or hospital showed that the respondents almost always have a conventional X-ray (99.05%) and an ultrasound machine (93.14%) available, but also diagnostics by means of CT imaging (87.43%) or magnetic resonance imaging (81.9%) is mostly immediately available.

Regarding their internal standards for imaging suspected upper ankle injury, 96% of participants indicated two-plane radiography, 27.05% indicated the use of Ottawa Ankle Rules, and 21.71% indicated ultrasonography.

In contrast, magnetic resonance imaging or computed tomography was cited as standard imaging by only 16.19% and 8%, respectively.

This was followed by several questions about the frequency of use of various diagnostic procedures. Here it was found that X-ray imaging in two planes is done always (16.38%) or in most cases (76.95%) by more than 90% of the respondents.

Ultrasound diagnostic, on the other hand, is rarely performed by 60%, in most cases by 19.81%, and always by 4.38%.

Ottawa Ankle Rules are used by more than 50% of the respondents always (20.76%) or in most cases (33.33%) and 29.14% rarely.

In contrast, it was indicated that CT imaging is rarely (88.57%) or never (7.24%) done, and MRI imaging examination is also rarely (89.33%) or never (2.67%) done by a large proportion of respondents.

However, if cross-sectional imaging is performed for suspected fractures, computed tomography is the preferred procedure by 55.62%, 35.24% prefer magnetic resonance imaging, and 9.14% prefer a combination of both procedures.

Among them, 61.9% use CT imaging for fracture progression analysis and 74.48% for surgical planning. 5.33% use it for 2-D reconstructions, 18.48% for 3-D reconstructions, and 22.86% for both 2-D and 3-D reconstructions.

Furthermore, 69.9% of participants report that their radiology department uses a pediatric protocol for CT exams, while 25.71% do not know if this is the case.

MRI imaging is used by 90.29% to detect concomitant injuries, by 24.57% for surgical planning, and by 20.38% for fracture progression analysis.

The majority of respondents (95.05%) considered cross-sectional imaging by CT or MRI useful for a triplane fracture, 59.24% for a twoplane fracture, 41.71% for a Salter-Harris type III or IV injury, and 8% for a Salter-Harris type I or II injury.

The survey ended with five questions asking for the participant’s own assessment regarding the indication for section imaging and the corresponding diagnostics on the basis of exemplary radiographs with ankle fractures in the age of adolescence.

**Fig. 1:**
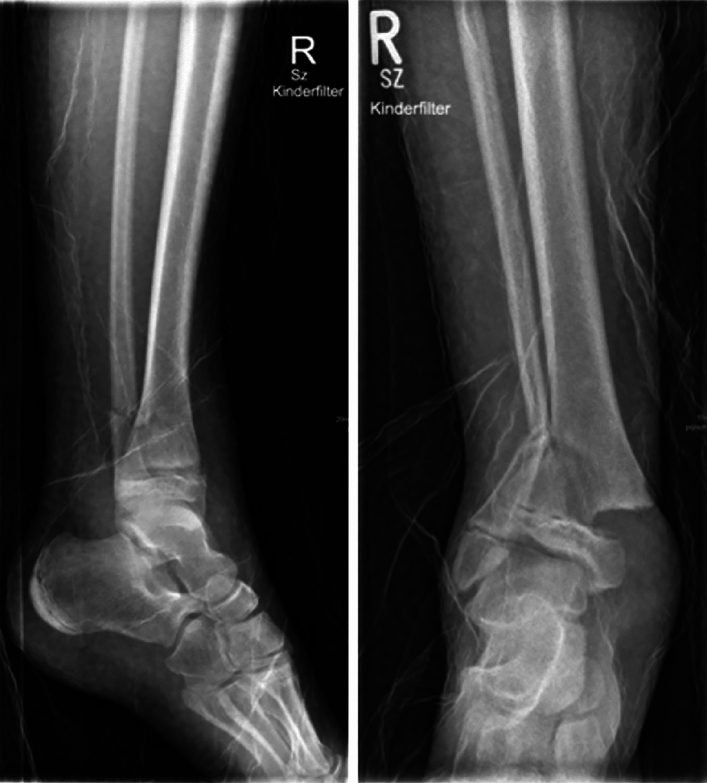
Case study 1 and 3

Case study 1 shows a fracture of the upper ankle joint type Aitken I/Salter-Harris II (Fig. 1). In this case, 51.43% of respondents indicated that they would not order cross-sectional imaging. 37.9% considered CT imaging and 6.67% MRI imaging to be indicated.

**Fig. 2:**
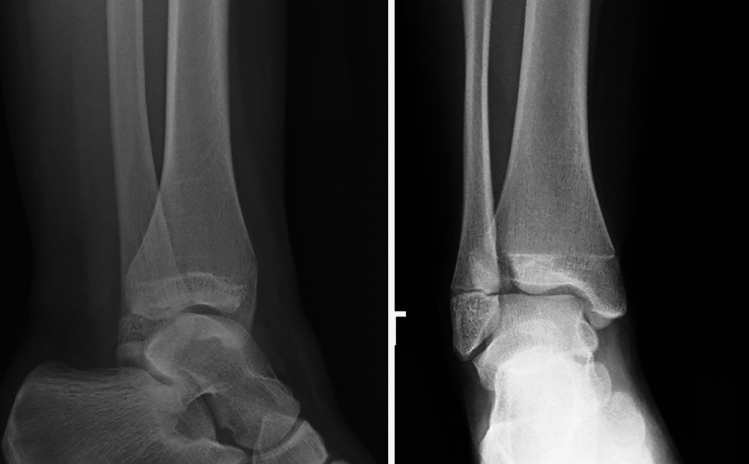
Case study 2

Case study 2 shows a triplane one fracture (in the x-ray imaging shown in the survey, the injury appears as a two plane fracture, the small metaphyseal fracture is only apparent on supplemental cross-sectional imaging) of the upper ankle (Fig. 2). For this injury, 40.19% of respondents would order CT imaging and 30.1% would order MRI imaging. 25.14% did not consider cross-sectional imaging to be indicated.

The figure in case study 3 was identical to that of case report 1. This was accidentally displayed again when editing the survey. However, here only 47.43% indicated that no cross-sectional imaging would be ordered. 40% consider CT imaging and 7.43% MRI imaging to be appropriate. This is roughly consistent with the information provided in question 17.

**Fig. 3:**
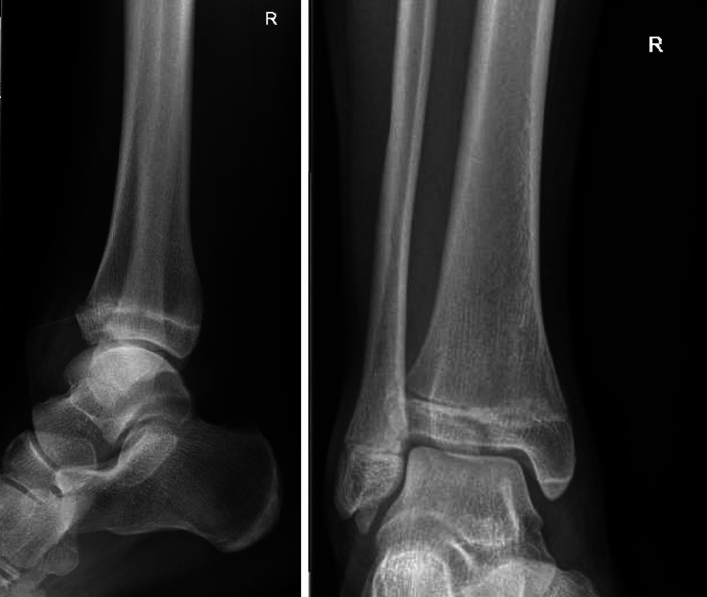
Case study 4

Case study 4 shows a two plane fracture of the upper ankle (Fig. 3). CT imaging is requested by 53.9% and MRI imaging by 21.52%. Only 22.67% do not consider cross-sectional imaging necessary.

**Fig. 4:**
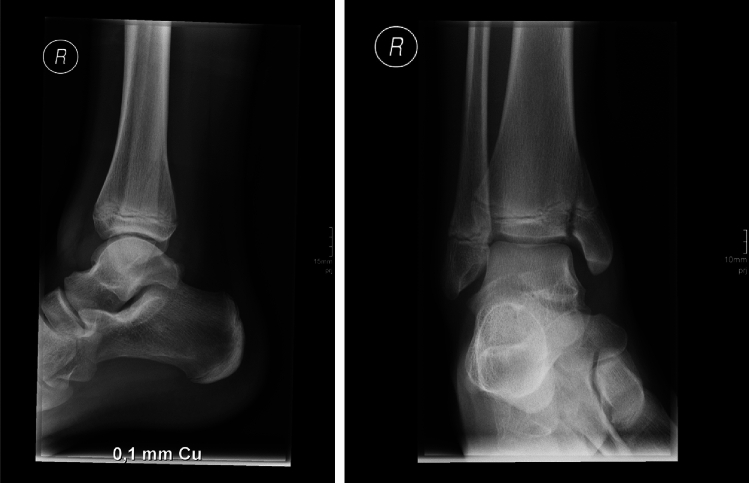
Case study 5

Case study 5 shows a fracture of the upper ankle joint type Aitken III/Salter Harris IV (Fig. 4). 65.33% of participants voted against cross-sectional imaging, 19.43% voted for CT imaging, and 13.9% voted for further diagnosis using MRI imaging.

## Discussion

The aim of this survey was to determine the current diagnostic procedure in Germany for suspected ankle fractures in children and adolescents.

The survey clearly showed that the conventional X-ray is still the gold standard and is used for primary diagnostics in the majority of cases (Table 1). Interestingly, the Ottawa Ankle Rules, which have also been well evaluated in childhood, are always or mostly used by more than 50% of the respondents, and ultrasound is regularly used by almost a quarter of the participants. A meta-analysis by Dowling et al. as well as other large studies, including one by Plint et al. have already shown that the application of the Ottawa Ankle Rules are validly transferable from adults to children and adolescents and can lead to a significant reduction in the number of necessary radiographs if applied consistently [10–12]. A reduction of 16–25% in necessary radiographs has been described [11, 12].

**Table 1:** Frequency of use of the following diagnostics for injuries of the distal lower leg in patients between 8 and 15 years of age

Frequency of use	Always	In most cases	Rarely	Never
Ultrasound	23 (4.38%)	104 (19.81%)	315 (60%)	83 (15.81%)
Ottawa-ankle-rules	109 (20.76%)	175 (33.33%)	153 (29.14%)	88 (16.76%)
Conventional x-ray	86 (16.38%)	404 (76.95%)	35 (6.67%)	0 (0.00%)
MRI scan	0 (0.00%)	42 (8.00%)	469 (89.33%)	14 (2.67%)
CT scan	2 (0.38%)	20 (3.81%)	465 (88.57%)	38 (7.24%)

Ultrasound diagnostics for the detection of infantile fractures is already an increasingly established method, especially in fractures of the distal radius and proximal humerus. In the latter, the working group led by Ackermann et al. was able to show that the number of necessary X-ray images can be reduced by up to 50% through consistent use [6]. However, the extent to which fracture sonography at the distal lower leg and upper ankle can replace radiographic imaging in the future or significantly reduce the number of images actually performed remains to be shown by further studies.

Furthermore, it was clear from this survey that there is an awareness of radiation safety in children in pediatric traumatology, although a quarter of all participants were unaware of the extent to which their radiology department has a specific pediatric protocol for CT imaging. Various studies from the past show that there is a strong endeavor to minimize radiation dose, especially in children and adolescents, and in addition to specially adapted pediatric protocols, the indication and extent of (emergency) CT imaging must be constantly critically scrutinized and adjusted accordingly [7, 13, 14].

Cross-sectional imaging is performed on a regular basis. In this context, the utility of CT and MRI imaging is constantly debated in the literature. While some authors consider two-level radiographic imaging to be sufficient in the majority of injuries and describe no change in subsequent therapy as a result of supplementary cross-sectional imaging in most cases, others consider supplementary CT or MRI diagnostics to be indicated in symptomatic clinical conditions despite unremarkable initial radiographic imaging and in intra-articular or dislocated fractures > 2 mm [1, 3, 15–17]. Our survey showed that CT imaging is mainly used for surgery planning and fracture course analysis (Table 2). MRI imaging, on the other hand, is mainly used to detect concomitant injuries, and only rarely for surgery planning or fracture progression analysis (Table 3).

**Table 2:** Reasons for CT imaging in fractures of the distal lower leg

Reasons for CT imaging	Responses
I never do a CT scan	44 (8.38%)
For correct classification	58 (11.05%)
For analysis of fracture course	325 (61.90%)
For surgery planning	391 (74.48%)
To detect concomitant injuries	100 (19.05%)

**Table 3:** Reasons for MRI imaging in fractures of the distal lower leg

Reasons for MRI imaging	Responses
I never do a MRI scan	20 (3.81%)
For correct classification	22 (4.19%)
For analysis of fracture course	107 (20.38%)
For surgery planning	129 (24.57%)
To detect concomitant injuries	474 (90.29%)

Complementary CT imaging with multiplanar reconstructions is often cited for the detection and accurate differentiation of triplane-1 and triplane-2 fractures and for a better understanding of the presenting injury pattern; here, particular reference is made to the more variable fracture course of triplane-1 fractures [18, 19]. It seems surprising to us that, according to our results, CT imaging was indicated more frequently in cases with twoplane than with triplane-1 fractures, which contradicts the results of the survey (Table 4).

**Table 4:** Injuries of the distal lower leg where participants consider cross-sectional imaging to be useful

Injuries where cross-sectional imaging is considered to be useful	Responses
Salter/Harris I + II	42 (8.00%)
Salter/Harris III + IV	219 (41.71%)
Two-plane fractures	311 (59.24%)
Triplane fractures	499 (95.05%)

Overall, with regard to the actual extent of applied (advanced) imaging in the context of this survey, a clear divergence between the queried theory and the actual practice becomes apparent, as it has already been presented in previous surveys and publications of Dresing et al. in SKT on the topic of imaging in children and adolescents [20–22]. Despite cautious and critical indications for cross-sectional imaging in theory, practice (illustrated in this work on the basis of the case studies) shows a much more generous and regular indication, especially with regard to radiation-relevant CT imaging. The clear ambition of all colleagues working in pediatric traumatology must be to raise awareness of and continuously optimize radiation protection, especially in children and adolescents.

It should be added that some of the respondents gave us feedback regarding cross-sectional imaging using digital volume tomography (DVT). This seems to be preferred over CT or MRI imaging in some clinics, also in children and adolescents. Unfortunately, in relation to pediatric joint injuries, there are no valid study results in the literature on the use of this imaging so far, but these would be desirable based on user feedback and subjective outcomes. It is possible that DVT is indeed a less radiation-intensive and thus more acceptable alternative to cross-sectional imaging in unclear cases for more accurate fracture visualization and delineation.

## Conclusion

In our opinion, sectional imaging by CT is too frequently performed in fractures of the distal lower leg with mostly stereotypical injuries, especially in two-plane fractures. Here, as in triplane-2 fractures, a stereotypical fracture course is usually seen [3]. Triplane-1 fractures, in contrast, show a certain variance; frequently, these injuries cannot be clearly distinguished on conventional radiographs, so supplementary cross-sectional imaging may well be considered here for accurate diagnosis. If the age of the patient and the possibilities in the working environment allow it, MRI imaging is preferable to computed tomography in our view because of the lack of radiation exposure and the possibility of assessing cartilaginous and ligamentous structures [8, 9].

In total a lot of cross-sectional imaging should be substituted by a deeper knowledge of pediatric injuries and their specific clinical and radiological features.
